# Greening African Cities for Sustainability: A Systematic Review of Urban Gardening’s Role in Biodiversity and Socio-Economic Resilience

**DOI:** 10.3390/plants14203187

**Published:** 2025-10-17

**Authors:** Philisiwe Felicity Mhlanga, Niké Susan Wesch, Moteng Elizabeth Moseri, Frank Harald Neumann, Nomali Ziphorah Ngobese

**Affiliations:** Unit for Environmental Sciences and Management, Faculty of Natural and Agricultural Sciences, North-West University, Private Bag X6001, Potchefstroom 2520, South Africa; nike.wesch@nwu.ac.za (N.S.W.); moteng.moseri@nwu.ac.za (M.E.M.); frank.neumann@nwu.ac.za (F.H.N.); nomali.ngobese@nwu.ac.za (N.Z.N.)

**Keywords:** biodiversity, climate adaptation, food security, green infrastructure, smart agriculture, sustainable development, urban planning

## Abstract

Urban gardening, particularly through food-producing green spaces, is increasingly recognized as a key strategy for addressing the complex challenges of climate change, food insecurity, biodiversity loss, and social inequity in African cities. This systematic review synthesizes evidence from 47 peer-reviewed studies across sub-Saharan Africa between 2000–2025 to analyze how urban home gardens, rooftop farms, and agroforestry systems contribute to sustainable urban development. The protocol follows PRISMA guidelines and focuses on (i) plant species selection for ecological resilience, (ii) integration of modern technologies in urban gardens, and (iii) socio-economic benefits to communities. The findings emphasize the ecological multifunctionality of urban gardens, which support services such as pollination, soil fertility, and microclimate regulation. Biodiversity services are shaped by both ecological and socio-economic factors, highlighting the importance of mechanisms such as polyculture, shared labour and management of urban gardens, pollinator activity and socio-economic status, reflected in sub-Saharan urban gardens. Socioeconomically, urban gardening plays a crucial role in enhancing household food security, income generation, and psychosocial resilience, particularly benefiting women and low-income communities. However, barriers exist, including insecure land tenure, water scarcity, weak technical support, and limited policy integration. Although technologies such as climate-smart practices and digital tools for irrigation are emerging, their adoption remains uneven. Research gaps include regional underrepresentation, a lack of longitudinal data, and limited focus on governance and gender dynamics. To unlock urban gardening’s full potential, future research and policy must adopt participatory, equity-driven approaches that bridge ecological knowledge with socio-political realities.

## 1. Introduction

Urbanization challenges environmental sustainability, biodiversity, and climate resilience. Replacement of vegetation and permeable soils with heat-absorbing asphalt, concrete, brick, glass, tiles, and metal [1] drives the urban heat island effect, resulting in increasing temperatures and stormwater runoff, and loss of habitats and biodiversity [2]. Land subsidence, groundwater contamination and flooding, especially in karstic landscapes, can arise [3]. Compromised urban air quality due to emissions of NOx, ozone, and particulate matter has become a significant concern for public health [4,5,6]. Anthropogenic climate change, with more frequent, intense heatwaves and storms, will exacerbate the problems of stormwater runoff, air-, water- and soil pollution, and urban heat [7]. The role of urban gardens, parks, urban forests, green roofs, permeable surfaces, wetlands, and green corridors is gaining attention [8,9]. Urban gardening in this review refers to the cultivation of food- and non-food plants in urban and peri-urban areas, using spaces managed for ecological, nutritional, or socio-economic benefits. These spaces include home, community, school and vertical gardens, rooftop farms, and small-scale urban agroforestry. In an African context, such gardens are embedded in diverse urban spaces, from densely populated informal settlements to sparsely planned residential neighbourhoods, ranging from household courtyards to community farm plots. This definition frames the scope of our analysis and ensures that both formally planned and informally established green spaces are considered within the broader category of urban gardening.

Urban green infrastructure encompasses the cultivation of ornamentals and food to enhance urban life and contributes to cooling, stormwater mitigation, groundwater recharge, pollutant removal, and biodiversity enhancement [10,11,12,13]. These benefits are contingent upon the appropriate selection of plant species or plant communities to maximize their effectiveness and avoid spreading of invader plants and insects [10,11]. Integrating food gardens into high-density developments might be challenging [14], necessitating inclusive urban planning on a regional level to ensure their multifunctionality [15]. By integrating ecosystem service functions, multifunctionality enables urban spaces to provide diverse societal and ecological benefits while addressing multiple challenges at once [16]. Potential ecosystem services must be considered: e.g.,

1. Urban gardening can pose environmental risks such as soil and groundwater contamination from heavy metals (e.g., lead, cadmium, arsenic) [17]
2. The green infrastructure can increase unwanted pest populations beyond economic damage thresholds if pest control is not well-managed [18].
3. The unwanted spread of weeds, invasive plant species or even a potential contribution to greenhouse gas emission [19].

Improper use of fertilizers and pesticides can also contribute to water pollution and biodiversity loss [19]. Plant selection is central to the success of urban gardens, yet current choices are often based on limited data or aesthetic preferences rather than ecological functionality [19,20,21,22,23,24,25,26,27,28,29]. The ecological and functional design of urban green infrastructure becomes even more critical in the context of rapid urbanisation and informality within African cities [30]. Africa’s urban population is expected to rise from 36% in 2010 to 50% by 2030 [31,32]. Urban expansion intensifies the demand for housing, transportation infrastructure, and energy [33] resources, frequently resulting in a reduction in green space [33], as urban planning systems often struggle to keep pace with the speed and scale of growth [34]. In addition to these systemic challenges, household reliance on fossil fuel combustion for cooking and heating within African low-income settlements contributes to both localized air pollution and elevated greenhouse gas emissions [35]. Rapid urbanization, weak planning, and limited funding exacerbate environmental stressors such as rising temperatures, poor air quality, and climate vulnerability [36].

In African urban centres, where food insecurity and vulnerability to climate change represent significant challenges, urban gardens and food-producing green spaces offer a promising avenue for building more resilient and sustainable food systems. This may involve the strategic integration of appropriate technologies to optimize productivity and resource efficiency in urban gardening practices, as well as the careful selection of plant species that are well-adapted to local environmental conditions and contribute to overall ecological balance. Furthermore, urban garden initiatives have the potential to empower local communities, generate economic opportunities, improve local food security and contribute to inclusive patterns of urban development, often referred to as urban farming. Nevertheless, the successful implementation of urban gardens in African cities necessitates careful consideration of several key factors. These include:

1. The selection of suitable plant species, prioritizing both spatial planning and the enhancement of biodiversity, with a focus on adaptation to local climatic and edaphic conditions.
2. The effective integration of urban gardens into broader urban planning and policy frameworks, ensuring their long-term viability, sustainability and scalability.
3. A thorough understanding of the prevailing socio-economic and cultural context, to ensure that urban garden initiatives are accepted, designed and implemented in a manner that maximizes accessibility and benefits for all urban residents.

**Figure 1:**
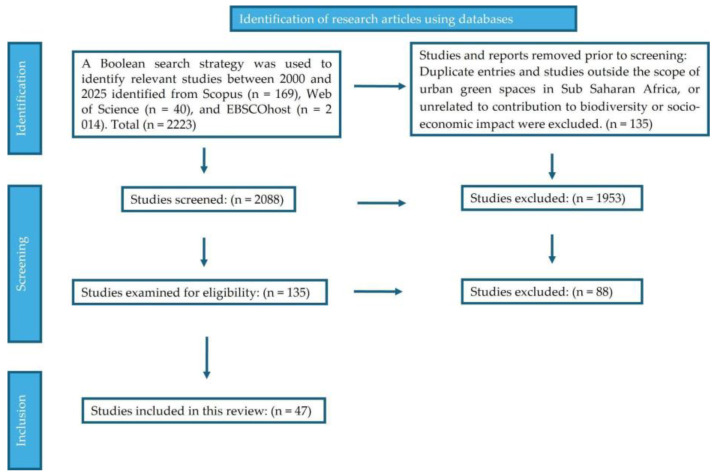
PRISMA flowchart of systematic selection process for literature at the identification and screening phase [39].

Research on African urban gardens mostly focuses on household food insecurity, poverty alleviation and broader urban food systems, but few look at the urban planning-food systems nexus [37,38]. This review seeks to systematically explore published knowledge on how food-producing green spaces contribute to addressing the interconnected challenges of environmental stress, including climate change, as well as food insecurity in African cities (Figure 1), from various perspectives, i.e., urban ecology, urban gardening, urban planning, urban resilience and sustainability.

## 2. Results

From the potentially relevant article, a total of *n* = 47 were selected for inclusion in this review (Figure 1).

### 2.1. Geography and Study Characteristics

A total of 47 studies were included in this systematic review, with a majority, approximately 65%, conducted within Sub-Saharan Africa, reflecting a strong regional focus. The most represented countries were South Africa (*n* = 18), Ethiopia (*n* = 4), Tanzania, Benin, Nigeria, Niger and Zambia (*n* = 3). Fewer studies originated from countries in Central Africa, such as Rwanda, Cameroon, and the Democratic Republic of Congo, indicating a geographical research gap in these regions.

**Figure 2:**
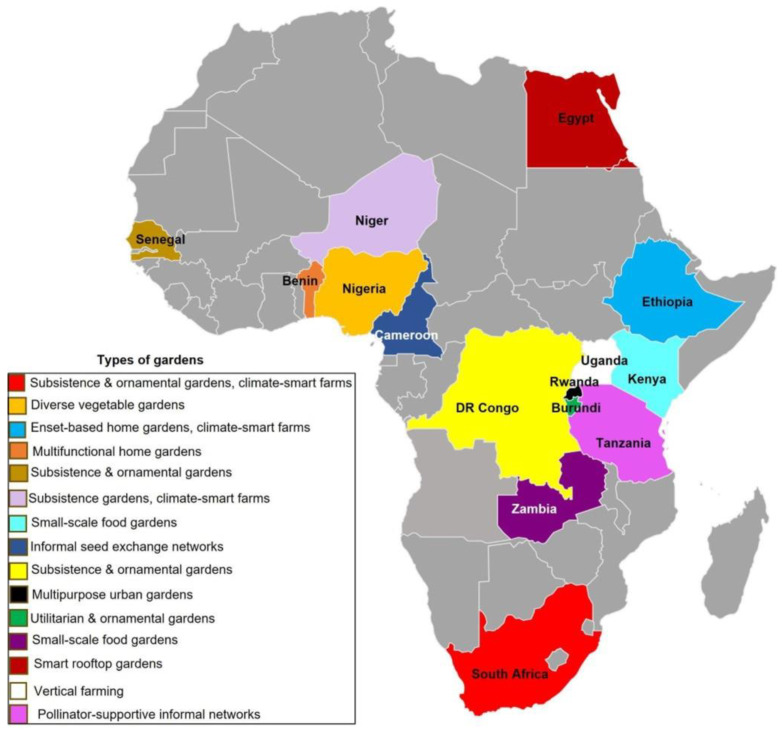
Types of urban gardens found in the regions reported in this review. The illustrated spaces include home gardens, community gardens, rooftop farms, vertical gardens, and small-scale urban agroforestry systems. Map template sourced and adapted from (https://www.pptmaps.com/Editable-map-of-Africa.html, Accessed 1 July 2025).

The predominant form of urban gardening explored across the studies was home gardens, followed by community gardens, rooftop gardens, school gardens, and urban agroforestry systems (Figure 2). These gardens were mainly situated in urban and peri-urban zones, often in low- to middle-income neighbourhoods.

In terms of methodological design, mixed-methods approaches were the most common, used in approximately 42% of the studies. These combined qualitative interviews, ethnographic surveys, and quantitative field measurements, enabling both socio-cultural insight and ecological assessment. Qualitative studies alone accounted for around 35%, primarily utilizing household interviews, focus groups, and case study narratives. A smaller portion, roughly 23%, employed purely quantitative designs, incorporating statistical modelling, biodiversity indices, and economic analyses.

Temporal coverage varied considerably across the dataset. While short-term studies lasting one to six months were common, especially for seasonal assessments and pilot projects, fewer studies (less than 15%) employed longitudinal or multi-year monitoring frameworks. This limited temporal depth underscores a need for future research tracking the long-term sustainability, ecological impacts, and policy integration of urban gardening initiatives.

Together, these geographic and methodological insights underscore the increasing scholarly and practical attention to urban gardening in Sub-Saharan Africa, while also revealing critical gaps in longitudinal data, regional coverage, and policy-oriented evaluation.

### 2.2. Plant Species Composition and Biodiversity

Plant species composition and biodiversity are central ecological attributes of urban gardening systems in Sub-Saharan Africa, contributing significantly to agrobiodiversity, ecosystem services, and cultural heritage. Across diverse contexts, urban gardens host a wide array of plant species, with species selection often influenced by environmental conditions, socio-economic factors, and functional needs such as food provision, medicinal use, income generation, and microclimate regulation. However, the criteria guiding species selection are inconsistently reported, highlighting a critical gap in understanding the ecological and socio-cultural rationale behind plant choices in urban contexts.

Garbole [40] conducted a comparative study of enset-based home garden agroforestry (EBHAF) and parkland agroforestry (PAF) systems in Ethiopia, revealing that EBHAF systems supported significantly greater species richness and ecological diversity. These gardens also outperformed PAF systems in terms of food production and sustainability indices. Land/space availability, which accounts for 80% of challenges encountered in EBHAF systems [40], can limit the success of polyculture systems. Similarly, Mellisse et al. [41] documented notable ecological degradation in Ethiopia’s Wondo Genet area following the shift from traditional polyculture gardens to khat (*Catha edulis*) monocultures. This shift was associated with declines in woody species richness, carbon stocks, and microclimate regulation, highlighting the ecological trade-offs of market-driven land-use decisions.

In Benin, Salako et al. [42] found the highest species diversity in home gardens situated within transitional climatic zones, with 285 species recorded across 240 gardens, including 20 crop wild relatives and 12 threatened taxa. These species served multiple purposes, particularly nutritional and medicinal. The work of Gbedomon et al. [43,44] extended these findings by examining how socio-demographic variables such as age, gender, and education shaped garden diversity. They noted that women tended to manage food-oriented gardens with higher species richness, while men were more likely to oversee gardens focused on medicinal plants. Jointly managed multifunctional gardens supported greater biodiversity, although the studies lacked a clear rationale for species selection, indicating a methodological gap in linking plant choice to functional or ecological outcomes.

Pollination services and biodiversity interactions were key themes in Tanzanian studies. Kingazi et al. [45,46] evaluated Chagga home gardens and reported high levels of alpha and gamma diversity among 293 woody species spanning 62 plant families. Key species such as *Grevillea robusta*, *Cordia africana*, and *Persea americana* were linked to increased pollinator activity and improved bean yields.

Southern African research also emphasized the influence of socio-economic and spatial dynamics on plant composition. Lubbe et al. [47] identified 835 plant species in 100 urban gardens in Potchefstroom, with a significant proportion being alien ornamentals, although many protected endemic species were also present. In Bushbuckridge, South Africa, High and Shackleton [48] observed a blend of wild and cultivated species, with each household typically using four to five wild plants and an average of 3.4 cultivated crops. These combinations contributed significantly to household nutrition and informal income. Further analysis across 450 households found tree diversity to be positively associated with suburb age and affluence, with older and wealthier suburbs hosting more diverse and denser tree populations [49]. Similarly, a transition toward ornamental alien species in affluent Batswana neighbourhoods was reported [50].

Cameroon offers an example of how informal networks maintain genetic diversity in urban settings. Rimlinger et al. [51,52] showed that urban residents often sourced seeds and seedlings from rural areas or local markets, fostering high intraspecific diversity. These networks were particularly important for culturally valued species like *Dacryodes edulis*.

In Senegal, research by Naigaga et al. [53,54] documented 96 plant species in Thies-region home gardens, spanning food crops, medicinal herbs, ornamentals, sacred plants, and species used for commerce. The data indicated that gardens serving multiple functions tended to have higher species richness, highlighting the connection between cultural practices and ecological outcomes.

Underutilized land remains a persistent challenge in parts of southern Africa. Kanosvamhira et al. [55] identified extensive tracts of urban land suitable for agriculture in Zimbabwe and South Africa yet found that low awareness and insufficient institutional support impeded its use. Related work by Kanosvamhira and Tevera [56] highlighted the struggles of informal gardening networks in Cape Flats, which were hindered by resource shortages and organizational fragmentation, limiting their resilience and impact.

Previous evidence from Niger, presented by Bernholt et al. [57] revealed that peri-urban gardening practices were influenced by ethnicity, garden size, and household composition. While commercial gardens were typically dominated by fewer high-value crops, subsistence gardens exhibited greater species diversity and a more balanced species distribution, emphasizing the socio-cultural factors shaping plant composition.

**Figure 3:**
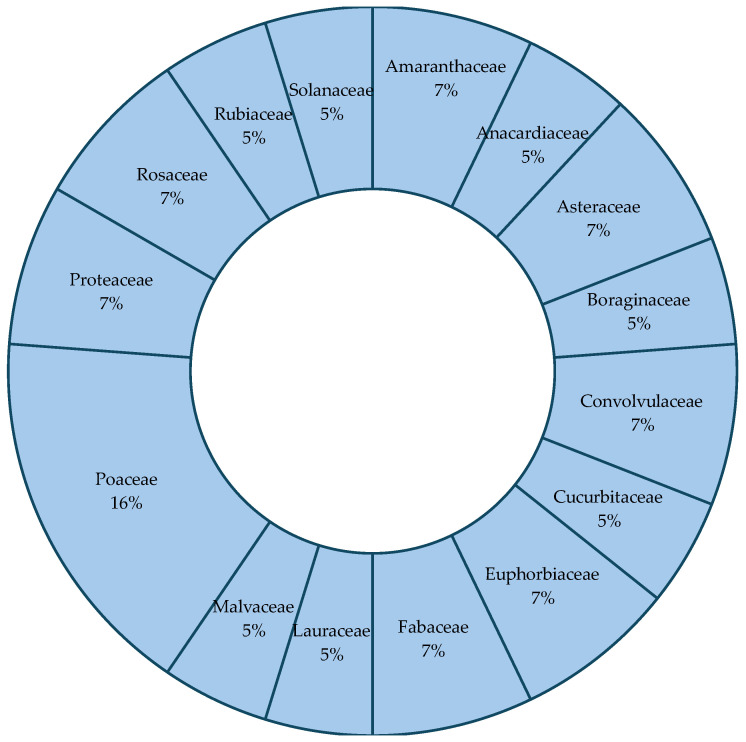
Relative frequency of the most cultivated crop families in urban gardens across African cities, based on the reviewed studies. The sunburst chart illustrates the proportional representation of crop families reported in home gardens, community plots, rooftop farms, and small-scale agroforestry systems.

To complement the qualitative synthesis of plant species diversity, we quantified the relative frequency of crop families reported across the reviewed studies. The chart (Figure 3) illustrates the predominance of Poaceae, Fabaceae, Euphabaceae, Proteaceae, Amaranthaceae and Asteraceae, reflecting their multifunctional roles in food production, medicinal use, soil fertility enhancement, and biodiversity support. The distribution highlights the prevalence of nutrient-dense, culturally significant, and ecologically functional crops in urban gardening systems, providing a quantitative synthesis to complement qualitative findings on plant diversity.

Despite the broad diversity of plants documented in urban gardens across Sub-Saharan Africa, many studies underreport the decision-making processes behind species selection. There is a growing need for research that explicitly connects species composition with ecological functionality, nutritional value, microclimatic benefits, and cultural relevance. More rigorous frameworks are required to analyze how urban gardeners select species based on local biophysical conditions, intended garden functions, and social contexts. Addressing this knowledge gap will enhance the capacity of urban gardens to support biodiversity conservation, climate resilience, and sustainable livelihoods.

### 2.3. Socioeconomic Impacts and Community Well-Being

Urban gardens across Sub-Saharan Africa play a critical role in strengthening food security, generating income, and enhancing community resilience, particularly for households in low-income and resource-constrained urban environments. These gardens are not only productive spaces but also social institutions that foster equity, knowledge-sharing, and environmental stewardship. However, the extent of their socio-economic benefits is mediated by contextual factors such as land tenure, gender roles, institutional support, and urban planning policies.

High and Shackleton [48], in 2000, provided quantitative insights into the economic value of residential gardens in South Africa’s Eastern Cape, estimating an annual contribution of R1694 (US$269) per household or R4200 (US$667) per hectare. Their findings showed that wild plant species made up 31% of the total garden value, while domesticated crops accounted for 69%, with the majority consumed by households and the remainder sold. This dual functionality highlights the role of urban gardens in both subsistence and informal economies.

Evidence from western Kenya, as captured by Hansen et al. [58], reaffirmed these socio-economic benefits. Through interviews with 30 households and 26 stakeholders, participants reported improvements in nutrition, financial relief, and women’s empowerment. However, the study also identified systemic challenges such as limited water availability, financial capital, land access, and technical support, suggesting that the benefits of gardening are heavily influenced by broader structural conditions.

Patterns observed in Ethiopia further support the association between demographic factors and garden productivity. Debie and Mengistie [59] found that adoption of urban gardening in Bahir Dar was significantly influenced by household size and residential plot size but limited by lower education levels. Complementing this, Garbole [40] documented high self-sufficiency among households using enset-based gardens, which produced *Zea mays* and *Phaseolus vulgaris* in quantities surpassing regional averages, thus supporting both crop and livestock farming.

In Benin, the relationship between garden structure and socio-demographic factors was made explicit by Gbedomon et al. [43,44], who found that garden diversity increased with age, education, and cooperative household management. Semi-arid regions hosted larger and more diverse gardens. Gender roles influenced garden functionality, with women often managing food gardens and men tending to medicinal plots. Importantly, joint management appeared to result in broader nutritional and economic outcomes.

Further south, socio-economic and historical contexts in South Africa have shaped gardening practices in unique ways. Research by Thamaga-Chitja et al. [60] showed that land ownership significantly reduced the likelihood of household food insecurity in KwaZulu-Natal. Other factors such as age, income, and cooperative membership positively influenced food security, while gender and market distance were associated with increased vulnerability. Khumalo and Sibanda [61] reported similar trends in Tongaat, also in KwaZulu-Natal, where 72% of urban gardening practitioners reported less food anxiety compared to 61% among non-practitioners, even though dietary diversity scores between the two groups did not differ significantly. Despite these promising outcomes, access to secure and formalized land remains a persistent challenge. Magidimisha et al. [62] noted that gardeners in Durban, South Africa, were often confined to informal or contested spaces, restricting access to credit and expansion opportunities. In Lusaka, Zambia, Simatele and Binns [63] attributed similar limitations to outdated policies and institutional fragmentation, even as urban gardening contributed meaningfully to food baskets.

The legacy of apartheid continues to influence perceptions and planning around urban gardening in South Africa. Thornton [64] argued that historical biases against subsistence farming have contributed to its marginalisation in urban policy frameworks. Nonetheless, studies by Lubbe et al. [47,65] and Bigirimana et al. [66] have shown that socioeconomic status shapes species selection, with wealthier communities favouring exotic ornamentals (e.g., *Mirabilis jalapa*, *Duranta erecta* and *Jacaranda mimosifolia*) and lower-income areas cultivating indigenous and edible plants (e.g., *Artemisia afra*, *Dovyalis caffra* and *Vigna unguiculata*), an ecological reflection of urban inequality because of historical injustice in the country.

Unequal access to green space is another key barrier. Kanosvamhira and Shade [67] reported that community gardens in lower-income areas of Cape Town often lacked institutional backing. Yet, these gardens continue to function as spaces of agroecological learning and food sovereignty. A related study by Kanosvamhira et al. [55] identified extensive tracts of highly suitable land that remain unused due to poor awareness and limited policy support, pointing to a missed opportunity for enhancing urban food systems.

Despite their diverse benefits, urban gardens face recurring barriers including limited water availability, insecure land tenure, lack of extension services, and weak policy integration. Moreover, while many studies acknowledge the importance of gender in shaping access and outcomes, few offer rigorous evaluations of how socio-cultural norms constrain or promote equitable participation in garden activities. Quantitative data on income generation, cost–benefit ratios, and long-term nutritional outcomes are also scarce, with most studies relying on self-reported perceptions or qualitative interviews.

In summary, urban gardens in Sub-Saharan Africa offer substantial yet uneven socio-economic gains. To fully harness their potential, future research must prioritise robust, longitudinal metrics, disaggregated by gender and income, and advocate for supportive governance frameworks that integrate urban gardening into formal development strategies. This will ensure that urban gardening not only alleviates food insecurity but also contributes to social justice and environmental sustainability.

### 2.4. Technological and Management Aspects

Urban gardening systems in Sub-Saharan Africa and other parts of the Global South are increasingly integrating climate-smart agricultural technologies (CSATs), though adoption remains uneven. These technologies offer a pathway to enhance productivity, resilience, and environmental sustainability, especially under growing climatic uncertainty, land pressure, and urbanization. This section synthesizes empirical evidence on the adoption, barriers, and performance of CSATs across different contexts.

In Nigeria, Mashi et al. [68] observed that awareness of CSATs among urban farmers in Kuje was positively associated with education, age, land quality, and income diversity, while traditional variables such as gender, religion, and institutional support had limited explanatory power. These findings highlight how climate-smart technology uptake may be conditioned more by knowledge and economic capital than by structural support.

Richter et al. [69], drawing on survey data from Nigeria, South Africa, and Zambia, found that despite high intentions to adopt smart technologies, barriers such as lack of trust, inadequate technical knowledge, and limited access to appropriate equipment constrain implementation. Interestingly, in Zambia, economic motivations and youth engagement were stronger drivers of uptake than institutional backing, whereas in South Africa, “responsibility diffusion” (i.e., concern that no one will take ownership) was cited as a major barrier. These findings suggest that the behavioural and social perceptions surrounding CSATs are as critical as material access.

In Ethiopia, Degefu and Kifle [70] explored on-farm and off-farm adaptations in response to climate variability among vegetable farmers in Addis Ababa. On-farm measures included water-smart practices like rainwater harvesting, nutrient-smart strategies such as green manure and intercropping, and knowledge-smart tactics such as switching crops and adjusting planting times. Off-farm adaptations included livelihood diversification and seasonal migration. This diversified response highlights the multifunctionality of CSATs beyond technical solutions, they represent knowledge systems embedded in broader socio-economic realities.

In South Africa, Khumalo et al. [71] reported that 74% of small-scale urban farmers in eThekwini Municipality were aware of CSATs, but only 66% used them at a moderate level. Common practices included crop rotation, mulching, drought-tolerant crops, and organic manure. The uptake was influenced by education, group membership, and farming experience, whereas household size and gender were negatively correlated with use. This points to the need for targeted extension support and farmer-to-farmer networks that account for gendered knowledge systems and resource access.

Masha et al. [72] further confirmed that CSAT-oriented urban gardening in Wolaita Sodo, Ethiopia, was significantly associated with food security improvements. Urban gardening adoption increased food security by 0.685-fold, with positive correlations to household size, training access, and market demand. These findings reaffirm the economic and nutritional potential of CSATs when appropriately contextualized.

Some electronic tools are beginning to scale in urban settings. Abdelhamid et al. [73] evaluated a solar-powered rooftop irrigation system in Egypt, demonstrating a 28.1% reduction in water and energy use, alongside a 28% reduction in carbon emissions compared to conventional systems. These findings present a compelling case for integrating renewable energy into urban food systems, though upfront costs and maintenance requirements remain potential barriers.

In West Africa, Tambol et al. [74] documented the ranking of climate-smart technologies among peri-urban smallholders in Niger. Biopesticides and organic manure were most used, followed by early-maturing and flood-tolerant cultivars. Tele-irrigation and green energy systems were also being trialled, but wider adoption was constrained by limited infrastructure and access to technical advisory services. Notably, these practices reflected strong adaptation to local climate risks, emphasizing the importance of aligning CSAT implementation with farmers’ lived experiences and environmental feedback.

Back in Ethiopia’s Gedeo Zone, Darge et al. [75] noted that smallholder farmers’ adaptation strategies centred on expanding home garden agroforestry systems, implementing modern practices, and maintaining traditional ecological knowledge. Their choices were shaped by age, education, climate risk exposure, and social networks. These results echo the need for integrated systems that leverage both indigenous and scientific knowledge frameworks.

In South Africa, recent work in the Gauteng Province emphasized the importance of water management training and extension services. Among the 1150 households surveyed, access to technical support significantly improved homestead gardening outcomes under drought conditions [76]. These findings advocate for investments in human capital and community engagement as levers for technological success.

Innovative vertical and rooftop farming is gaining traction. Lawrence and Jeeva [77] evaluated the Priority Zone Rooftop Garden in Durban, showing that despite small spatial footprints, rooftop gardens provide food, training, and employment while reducing infrastructure decay. Similarly, Ngie and Sithole [78] noted that hydroponic farming in Johannesburg city centre utilized abandoned spaces efficiently but required more robust tenure policies and infrastructural support for scale-up.

Gumisiriza et al. [79] tested a vertical soilless hydroponic system in Uganda, estimating a return on investment of over 12% for small-scale lettuce production. Their cost–benefit analysis indicated that such systems, if expanded and supported, can contribute to sustainable urban food production, though sensitivity to input prices and market access must be considered.

Comparative analyses between Kigali, Rwanda, and Singapore showed divergent trajectories. While Singapore favoured high-tech, commercial-scale smart agriculture, Kigali maintained a grassroots-led model where unused urban spaces were converted into productive gardens [80]. These findings suggest that local governance models and urban form strongly shape the technological pathways adopted.

Taken together, the evidence from the reviewed studies reveals a dual challenge: while CSATs are increasingly recognized as pivotal to sustainable urban food systems, their success hinges on more than just technical efficacy. Socioeconomic inequalities, institutional readiness, and access to training continue to dictate which communities can adopt and benefit from these innovations. Future work should explore participatory technology co-design, harmonisation with indigenous practices, and financing models that mitigate the risks associated with adoption among low-income urban farmers.

### 2.5. Urban Gardens and Planning

Urban planning and governance considerations were notably underrepresented in the reviewed literature. In recent years, gardening, particularly in the form of urban gardening, has gained recognition across Africa for its contribution to socio-economic development and urban greening [38,81,82]. However, its integration into spatial planning policies remains inconsistent and fragmented. In many African cities, urban gardening is conducted informally, often without legal recognition of land ownership, which undermines its long-term sustainability. For instance, while South Africa has shown some appreciation for urban gardening, it still lacks a comprehensive national framework to formally incorporate it into urban planning [83]. This often results in self-initiated gardening projects being undervalued and regarded as temporary land uses.

Some municipalities, such as those in Nelson Mandela Bay and Cape Town, have taken steps to address this policy gap by developing urban gardening strategies that encourage small-scale urban farming [37]. Despite these promising developments, the systematic integration of gardening activities into urban spatial plans continues to face challenges related to limited funding, entrenched attitudes, and a lack of robust evidence to justify its inclusion. Nevertheless, urban gardening holds significant potential to inform more structured and inclusive urban development policies that enhance sustainability, resilience, and social equity.

In Lagos, Nigeria, Awoniran et al. [84] illustrated how urban expansion has considerably reduced land available for agriculture. Between 1986 and 2016, over 47 percent of urban farmland was lost, with 75 to 89 percent of cultivated land converted to other uses. The authors proposed strategic zoning policies that designate buffer areas along infrastructure corridors, such as roads, streams, and power lines, for protected urban farming. These recommendations highlight how spatial planning can contribute to safeguarding urban food systems and enhancing ecological infrastructure.

Similarly, in Lusaka, Zambia, Simatele et al. [85] investigated how climate change and urban governance intersect to influence small-scale farming. Their findings revealed that although urban gardening is central to household livelihoods, it is vulnerable to extreme weather events and hindered by weak institutional coordination. The study emphasized the need for deliberate and inclusive planning to strengthen the resilience and sustainability of urban gardening in the face of climate variability.

Only a small number of studies explicitly examined the intersection of urban gardening and formal planning frameworks. However, several others addressed themes highly relevant to urban planning, including climate resilience, strategic land use, community-based food systems, technology-driven planning, and institutional support structures. Among those studies, the extent and framing of engagement with planning concepts varied. For example, Richter et al. [69], using data from Nigeria, South Africa, and Zambia, found that institutional trust and perceived community benefit were key drivers for adopting smart urban farming practices. Their findings underscore the importance of localised governance and community participation in planning for urban gardening.

Masha et al. [72] and Khumalo et al. [71] similarly called for integrated municipal support systems to scale up climate-smart agricultural practices in urban settings. Yet their work also highlighted enduring governance barriers, including insecure land tenure, fragmented zoning laws, and institutional inefficiencies that obstruct the mainstreaming of urban gardening into city development frameworks. Bigirimana et al. [66] offered further evidence from Burundi, showing that socio-economic disparities and urban morphology significantly influence plant diversity and garden type. Their analysis implied that spatial planning and governance structures, though often overlooked, are instrumental in determining the accessibility, function, and sustainability of urban gardens.

Taken together, the reviewed studies suggest that while urban gardening is widely practiced and increasingly valued, its integration into urban development frameworks remains weak. Institutional inertia, land-use competition, and the absence of evidence-based planning tools continue to marginalize gardening initiatives. Nonetheless, the literature also reveals actionable pathways forward, such as zoning land for green infrastructure, engaging communities in participatory planning, and developing enabling policies. These steps could elevate urban gardening as a core strategy in promoting sustainability, inclusiveness, and resilience in African cities.

## 3. Discussion

This systematic review of 47 studies offers a better understanding of the role of urban gardening in enhancing climate resilience, food security, biodiversity conservation, and socio-economic sustainability across African cities. The results underscore urban gardening as a multifaceted tool for sustainable development, whose potential can be fully realized only when integrated into urban policy, supported by appropriate technologies, and inclusive of socio-cultural dynamics.

### 3.1. Geographical Representation and Methodological Insights

The geographical distribution of studies reviewed in this systematic analysis indicates a notable regional concentration, with approximately 65% of the 47 included studies conducted in sub-Saharan Africa. South Africa [47,48,49,50,55,56,60,61,62,64,65,67,69,71,76,77,78,86], Ethiopia [40,41,59,72,75], Tanzania [45,46], Benin [42,43,44] and Nigeria [68,69,84], emerged as the most represented countries, reflecting the growing scholarly and institutional interest in urban gardening in these contexts. However, this distribution also exposes a marked underrepresentation of studies from Central Africa, including countries such as Rwanda, Cameroon, and the Democratic Republic of Congo. The limited empirical data from these regions presents a substantial gap in the better understanding of urban gardening across diverse agroecological, socio-political, and economic environments.

This regional skew may reflect disparities in research infrastructure including better publication channels and funding availability, or differing levels of policy recognition and integration of urban gardening. For instance, the relatively high volume of research in South Africa and Ethiopia may be attributed to more established and financially stronger academic institutions, donor interest, or municipal-level urban gardening initiatives, whereas other regions remain underexplored due to limited institutional support or restrictive urban planning regimes. Addressing this imbalance will require deliberate regional engagement and inclusive research collaborations that incorporate neglected geographies. Yet, this imbalance also raises a paradox: conclusions from South Africa or Ethiopia are often generalized across the continent, even though ecological and socio-political realities in Central Africa differ markedly.

In terms of the methods, the dominance of qualitative (35%) and mixed-methods (42%) approaches highlights the field’s emphasis on capturing the socio-cultural, behavioural, and participatory aspects of urban gardening. These approaches are valuable in elucidating community experiences, cultural norms, and informal governance mechanisms that underpin garden design and management. However, their prevalence also points to a recurring limitation: the relative scarcity of quantitative and longitudinal studies capable of capturing biophysical trends, economic returns, and long-term impacts. Only 23% of studies adopted purely quantitative frameworks, and less than 15% employed extended temporal coverage. Furthermore, there is a lack of methodological standardization across studies, which complicates the measurement of biodiversity indices, ecosystem services, or food security outcomes, thereby affecting the comparability and reliability of research findings. This variability complicates efforts to synthesize data or conduct cross-regional comparisons. It also limits the scalability of successful models or the development of urban gardening typologies applicable across different African urban contexts. A tension therefore persists between rich qualitative insights that highlight lived experiences and the need for standardised metrics that can substantiate claims of resilience and food security at scale.

Future research would benefit from an expansion of longitudinal and spatially explicit studies, including the use of Geographic Information Systems (GIS), remote sensing, participatory mapping and virtual reality technology. While there are no reported examples of the use of virtual reality in Africa, such technologies simulating 3-D spaces can be explored to enhance the planning and development of urban gardens [87]. Overall, while the reviewed literature demonstrates growing academic attention to urban gardening in Sub-Saharan Africa, addressing the geographic and methodological gaps remains critical to enhancing the scientific rigor and policy relevance of future research.

### 3.2. Biodiversity and Ecological Functionality

Urban gardens across Sub-Saharan Africa serve as vital reservoirs of plant biodiversity and providers of essential ecosystem services, particularly in densely populated and ecologically stressed urban environments [88]. The reviewed studies consistently illustrate that gardens with high species richness enhance ecological functionality by providing services such as carbon sequestration, pollination support, soil nutrient cycling, and microclimate regulation. These ecological contributions are most evident in gardens that incorporate a diversity of woody perennials, herbaceous crops, and multifunctional species adapted to local conditions.

Enset-based home garden agroforestry systems (EBHAF) supported significantly higher plant species richness and ecosystem productivity compared to parkland agroforestry systems, with food yield and sustainability indices exceeding the Organisation for Economic Co-operation and Development (OECD) thresholds [40]. The results corroborate previous research indicating that polyculture systems significantly contribute to the enhancement of soil fertility, agrobiodiversity, and household food security [89]. Similarly, Chagga home gardens demonstrated substantial alpha and gamma diversity, encompassing 293 woody species. Notably, species such as *Grevillea robusta*, *Cordia africana*, and *Persea americana* were important in augmenting pollinator visitation rates and enhancing crop productivity [46]. This illustrates how ecological and agronomic goals can be aligned through intentional species selection.

In Benin, multifunctional gardens hosting both food and medicinal plants had higher biodiversity indices [42,43,44], supporting the view that multifunctionality, rather than monocultural specialization, underpins agroecological resilience [89]. However, the ecological integrity of urban gardens can be undermined by market-driven land-use transitions. For instance, Mellisse et al. [41] reported that in Wondo Genet, Ethiopia, the replacement of diverse home gardens with monocultures of *Catha edulis* led to marked reductions in species richness, carbon biomass, and microclimatic buffering. This report is consistent with previous studies in Ethiopia on the loss of soil organic carbon stock due to the replacement of coffee-based home gardens with khat monoculture systems [90,91]. These findings expose the inherent trade-offs between short-term economic incentives and long-term ecological sustainability, highlighting a key debate in the literature about whether market forces inevitably erode biodiversity in urban gardens.

Case studies from South Africa provide additional insights, demonstrating that socio-economic gradients influence biodiversity outcomes. Gardens combining wild and cultivated species contributed to both nutritional diversity and household income [48]. There was a considerable coexistence of alien ornamental plants and protected indigenous species across 10 sampled gardens in Potchefstroom [47]. Kaoma and Shackleton [49] found that wealthier, older suburbs maintained greater tree diversity, while low-income areas had less vegetation cover. This observation potentially indicates that socio-economic status frequently supersedes cultural traditions in determining garden composition, while affluence and strategic planning influence ecological configurations. These variations point to unresolved debates over whether cultural traditions or socio-economic gradients are the primary determinants of biodiversity outcomes in urban gardens.

In addition to species richness, informal seed exchange networks play an important role in enhancing genetic diversity and promoting intraspecific conservation, as demonstrated by Rimlinger et al. [51,52] in their studies conducted in Cameroon. Urban gardeners often source seeds from rural communities or kinship networks, which fosters high genetic variation and the conservation of culturally significant species such as *Dacryodes edulis*. This dynamic emphasizes the role of urban gardens in conserving both agrobiodiversity and traditional ecological knowledge.

Despite these contributions, many studies underreported the selection criteria for plant species, limiting our understanding of how ecological functionality is intentionally planned or emergent. While some species are chosen for their cultural or nutritional value, few studies explicitly addressed traits such as drought tolerance, pollinator attraction, or carbon sequestration potential. This limitation hinders the widespread adoption and adaptation of effective models of climate-resilient urban horticulture across diverse environmental contexts.

To enhance the ecological role of urban gardens, future research must adopt a trait-based, context-sensitive approach that links plant selection to ecosystem service delivery. Urban greening strategies should prioritize native and climate-resilient species and support multifunctional garden designs that reflect both ecological and socio-cultural priorities. Bridging scientific knowledge with local practices and participatory planning will be essential for integrating biodiversity conservation into the broader sustainability and climate resilience agendas of African cities.

### 3.3. Community Resilience and Socioeconomic Implications

This review reveals consistent associations between gardening and improvements in food security, income diversification, and psychosocial well-being, especially among low-income households. The socio-economic benefits of urban gardening are not uniformly distributed but are shaped by household characteristics, tenure arrangements, and the broader urban governance landscape. Gender and age played recurring roles in determining garden use and management structure. While women predominantly stewarded subsistence gardens, often as primary caregivers and food providers, men were more involved in cultivating medicinal and commercial species. However, when gardens were managed jointly, especially in multifunctional systems, diversity and utility were improved, emphasizing the role of equitable household dynamics in reinforcing socio-economic resilience [43,44].

Yet, there are contradictions in the literature about the extent of these benefits. Some studies report substantial contributors of gardens to household income and dietary diversity [48], while others caution that their outputs are often too limited to meaningfully reduce vulnerability, particularly in contexts of deep structural poverty [62,67]. Similarly, while gardens are frequently framed as empowering for women, this narrative sometimes obscures the persistence of gendered labour burdens and limited decision-making power in resource allocations. These contrasting accounts suggest that urban gardening may simultaneously reinforce and challenge existing inequalities depending on local contexts.

Another unresolved debate concerns the valuation of socio-economic outcomes. Some authors emphasize psychosocial benefits such as improved well-being, social cohesion, and intergenerational knowledge transfer [43], whereas others argue that without quantifiable data on income or nutritional outcomes, these ‘soft benefits’ risk being undervalued by policymakers [48]. This represents a missed opportunity for substantiating the economic case for urban gardening within city development agendas.

Scalability also remains contested. While some case studies highlight the potential of gardens to buffer households during shocks such as the COVID-19 pandemic, but without structural support, such as secure land tenure, credit and extension services [62,63,67], urban gardening will remain marginal and unable to transform urban food systems. These debates underline the need for empirical studies that move beyond celebrity narratives to quantify and contextualize resilience outcomes across different socio-economic settings.

Despite these tensions, the evidence converges on the importance of targeted interventions. Urban gardens function not only as sites of food production but also as loci of community cohesion, intergenerational knowledge transfer, and environmental education. In this sense, gardens act as adaptive social-ecological systems, capable of absorbing shocks while delivering essential services. For urban gardening to move beyond its current marginalised position, targeted policy interventions are essential. These include securing land tenure, improving access to water and inputs, strengthening extension services, and embedding gender-equity principles into programme design. To address these challenges, future research should prioritize disaggregated, empirical data on nutritional outcomes, income generation, and household resilience, thereby building a more persuasive evidence base for integration into urban planning and food policy frameworks.

### 3.4. Technological Integration and Sustainable Management

The integration of climate-smart and resource-efficient technologies into urban gardening practices highlights the importance of addressing environmental constraints and achieving sustainable intensification, as demonstrated by various adaptive techniques and innovations. Across sub-Saharan Africa, small-scale gardeners commonly employed adaptive techniques such as mulching, composting, intercropping, and the use of drought-tolerant plant varieties, which enhance soil health, improve water efficiency, and stabilizes yield, thereby contributing to the integration of sustainable technologies. In Egypt and South Africa, more advanced innovations, including mobile-app-controlled irrigation systems and vertical gardening [71,73,77,78], demonstrated tangible reductions in water use, energy consumption, and carbon emissions, underscoring their relevance for integrating sustainable technologies into urban garden practices.

The literature also highlights tensions between low-cost, indigenous methods and high-tech solutions. While some scholars argue that simple, low-input practices are the most context-appropriate and scalable in resource-constrained settings, others emphasize that digital technologies such as precision irrigation or vertical systems offer long-term efficiency gains. This debate reflects broader concerns about equity: technologies that reduce water use may be desirable, but their adoption is often limited to wealthier households or pilot projects supported by Non-Governmental Organizations (NGOs) leaving poorer communities dependent on more traditional, labour-intensive practices.

Despite the promising developments in technological innovations, their adoption remains uneven and is highly dependent on socio-economic factors, access to technical knowledge, and the presence of functional extension services. Some studies report that uptake is primarily constrained by cost and infrastructure [71,73], whereas others highlight behavioural barriers, such as ‘responsibility diffusion’, where uncertainty about who should lead technological implementation undermines collective action [69]. Furthermore, many technological interventions are not sufficiently tailored to local ecological and cultural contexts, raising concerns about their long-term sustainability and relevance across diverse African cities.

To bridge the gaps in adoption and ensure long-term sustainability, future strategies must prioritize participatory technology development that integrates indigenous knowledge systems with modern innovations. Localized research, peer-to-peer learning platforms, and inclusive capacity-building initiatives are essential to align technological solutions with the needs and constraints of diverse urban gardeners. Ultimately, sustainable urban gardening must be knowledge-intensive, climate-adaptive, and socially inclusive to support long-term ecological and food system resilience. Reconciling the divide between low-cost, accessible practices and advanced technological interventions will be central to ensuring that innovations serve all urban gardeners rather than deepening existing inequalities.

### 3.5. Urban Planning and Governance Challenges

Urban gardens, despite their recognized ecological and socio-economic value, remain largely marginalized in formal urban planning and governance frameworks across sub-Saharan Africa. Most urban gardening initiatives operate informally, without secure land tenure, institutional support, or integration into municipal development strategies. In the reviewed papers, only a handful of cities, such as Cape Town and Kigali, have taken steps to embed urban gardening within broader urban sustainability and land-use planning efforts. The lack of integration of urban gardens into formal planning frameworks represents a critical governance gap. Studies by Richter et al. [69] and Masha et al. [72] reveal that institutional credibility, municipal support, and community trust are key determinants of successful urban gardening implementation. Conversely, the lack of clear zoning policies, secure land rights, and enabling legislation undermines the scalability and long-term viability of urban gardening. In many cities, land allocated for agriculture is vulnerable to development pressures, and the lack of participatory planning mechanisms excludes gardeners from decision-making processes.

Moreover, socio-economic gradients in garden diversity, observed in contexts such as South Africa and Burundi, highlight the indirect influence of urban design and neighbourhood-level planning on ecological outcomes, further complicating governance challenges. Integrating urban gardens into green infrastructure networks and climate resilience strategies could significantly enhance both biodiversity and social equity. To unlock the full potential of urban gardening, governance structures must evolve beyond ad hoc initiatives. This requires institutionalizing urban gardening through policy reforms, secure and equitable land tenure arrangements, and alignment with national climate adaptation and food security goals. Investments in public education, community-based planning, and multi-level coordination between local and national governments are also vital. When effectively supported and integrated, urban gardening can serve as a cost-effective and transformative solution to intersecting challenges such as climate change, food insecurity, and urban inequality. As such, it should be considered a foundational pillar of sustainable and inclusive urban development in the Global South.

### 3.6. Limitations and Research Gaps

**Figure 4:**
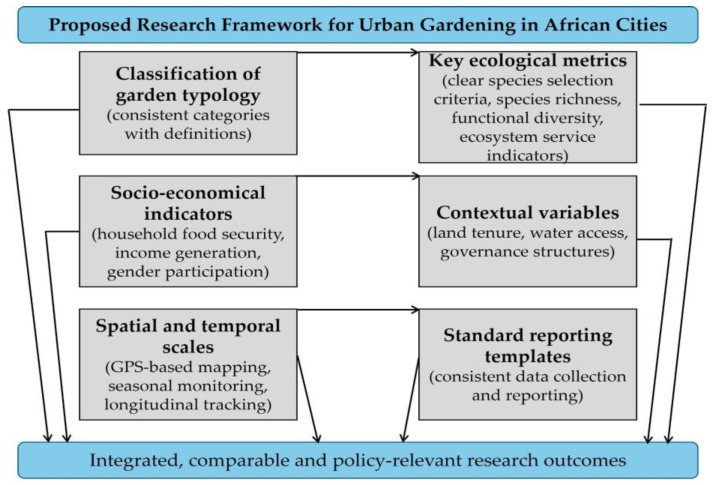
Proposed research framework for the standardization of urban gardening reporting in African cities.

Although this review highlights the different benefits of urban gardening, it also notes the limitations and persistent research gaps that limit understanding and effective policy formulation. Firstly, there is a significant geographical bias in the existing literature. A disproportionate number of studies are concentrated in a few countries, such as South Africa, Kenya, and Nigeria, while other sub-Saharan regions, particularly in Central Africa and smaller urban centres, remain underrepresented. This uneven coverage limits the generalizability of findings across the diverse socio-ecological and political contexts of the Global South. Secondly, methodological heterogeneity across studies, ranging from purely qualitative case studies to limited-scope quantitative surveys, impedes robust cross-comparison. Few studies employed longitudinal designs or mixed-method approaches capable of capturing the dynamic and seasonal nature of urban gardening practices, resilience outcomes, or long-term sustainability. Additionally, standardized metrics for evaluating ecosystem services, biodiversity contributions, or climate adaptation outcomes are lacking, making it difficult to assess and compare performance across settings. Thirdly, there is limited exploration of the interlinkages between urban gardening and broader governance, technological, or economic systems. While some studies touch on issues such as land tenure or climate-smart practices, few provide in-depth analyses of institutional frameworks, financial mechanisms, or market linkages that enable or constrain urban gardening. Similarly, the gendered dimensions of participation, decision-making, and benefit-sharing, though frequently mentioned, require more rigorous intersectional analysis. Moreover, the socio-cultural dimensions of urban gardening, such as indigenous knowledge systems, aesthetic values, and cultural ecosystem services, remain largely overlooked in favour of utilitarian and productivity-focused assessments. Focusing solely on utilitarian and productivity-focused assessments risks excluding critical local perspectives that shape gardening practices and their societal acceptance. To address the current lack of consistency in urban gardening research in African cities, we propose a standardized research framework (Figure 4) that integrates garden typology, ecological metrics, socio-economic indicators, and contextual variables. Such a framework will enhance cross-study comparability, enable evidence-based policy design, and provide a replicable methodology for future urban gardening initiatives. From a policy perspective, our synthesis of crop family diversity offers a reference point for developing urban gardening plant selection guidelines that balance nutritional value, ecological resilience, and cultural relevance. Municipal and national policies could integrate these insights into urban gardening strategies, ensuring that promoted plant lists reflect both community needs and biodiversity conservation goals.

Future research should therefore prioritize: (1) expanding geographical and urban typology coverage; (2) employing standardized, interdisciplinary methodologies; (3) investigating institutional, economic, and governance mechanisms; (4) mainstreaming gender and social equity analysis; and (5) exploring under-researched dimensions such as cultural identity, mental health, and intergenerational knowledge transfer. Addressing these gaps is crucial for developing inclusive, evidence-based urban gardening policies that unlock the full potential of urban gardening to support climate resilience, food sovereignty, and social justice in developing countries.

## 4. Materials and Methods

### 4.1. Systematic Literature Review

A structured, multi-phase systematic literature review was conducted to explore the role of urban gardening in addressing climate-related and food insecurity challenges in sub-Saharan Africa. This review emphasizes three key aspects:

(i) selection of plant species for their adaptation to the local environmental conditions, enhancing biodiversity and ecological resilience, (ii) integration of modern technologies, e.g., irrigation, in urban garden systems, and (iii) socio-economic impacts and contributions to community resilience and health. In addition to ecological and agronomic dimensions, this review includes studies from a city and town planning perspective to evaluate how urban gardens are integrated into broader urban development frameworks, land-use strategies, and climate adaptation planning. The Preferred Reporting Items for Systematic Reviews and Meta-Analyses (PRISMA) guidelines [38] were employed to ensure transparency, replicability, and methodological rigour across all stages of the review. The process followed four structured phases used as keywords when conducting a literature search: identification, screening, eligibility assessment, and inclusion. The protocol followed was registered on protocol.io (https://www.protocols.io, Accessed on 22 July 2025).

#### 4.1.1. Identification

Relevant peer-reviewed articles and grey literature (government reports, technical documents, dissertations, and urban development plans) published between 2000 and 2025 were sourced from multiple databases: Scopus, Web of Science and Urban Studies Abstracts (EBSCOhost). The search employed a Boolean strategy combining keywords related to urban gardening, climate change, food security, sustainable development, and urban planning (Table 1). To ensure an inclusive and interdisciplinary scope, search terms were selected to capture both ecological/agronomic studies and those rooted in urban planning, spatial development, or municipal governance. This allowed the review to include city-scale interventions, case studies on community gardens, urban gardening zoning policies, and participatory design initiatives that position urban gardening within broader sustainability and resilience frameworks. Duplicate records and studies falling outside the thematic or regional scope (e.g., studies focusing solely on rural agriculture or conducted in non-developing country contexts) were excluded at this stage.

#### 4.1.2. Screening

The screening process commenced with a preliminary assessment of article titles and abstracts to determine their relevance to the review’s objectives. Articles were shortlisted for full-text review if they met at least one thematic area (climate mitigation, food security, or planning integration) and demonstrated empirical grounding. Studies that examined the role of urban gardens as instruments within formal city planning or community development strategies, including participatory urban design, zoning frameworks, or climate adaptation plans were also included. To maintain the integrity of the selection process, duplicate records were removed, and studies that were non-peer-reviewed, published in languages other than English, or inaccessible through institutional databases were excluded.

#### 4.1.3. Eligibility

A set of predefined eligibility criteria was applied to ensure the inclusion of high-quality, relevant studies. Studies were assessed based on their methodological rigour, relevance to African urban gardening, and direct contribution to socio-economy and climate change mitigation. The following inclusion criteria were used:

1. Publication type and language: Only studies published in peer-reviewed English-language journals or grey literature (including government and technical reports, theses, dissertations, and conference proceedings) between 2000 and 2025 were considered.
2. Topical relevance: The study must examine urban gardens in the context of climate change mitigation, biodiversity, food security, or city/town planning.
3. Geographical relevance: The study should be based in or include case studies from sub-Saharan Africa.
4. Empirical evidence: Eligible studies must present field data, observational case studies, or documented planning interventions, rather than theoretical modelling alone.
5. Analytical rigour: The included studies provide statistical analysis, policy evaluation, spatial planning models, or community-level outcome assessments.

Studies were excluded if they:

(a) Were solely descriptive without analytical depth.
(b) Focused exclusively on rural agriculture without urban context.
(c) Lacked evidence of real-world application or planning impact.
(d) Addressed general sustainability topics without specific reference to urban gardening.
(e) Focused on controlled greenhouse or laboratory experiments unless field validation was also provided.

#### 4.1.4. Inclusion

A final list of eligible studies was compiled based on the above criteria. Each study was logged using a custom-built Excel matrix that documented location, year, urban garden type, plant species used, reported outcomes, and urban planning dimensions (e.g., zoning support, land-use integration, and policy alignment). Additional planning metrics, such as participatory governance, accessibility, and municipal support, were also coded where available to better understand the intersection between urban gardening and city design.

### 4.2. Data Analysis

Data extracted from the eligible studies were systematically organised into thematic categories to facilitate structured synthesis and comparison. The analysis focused on identifying patterns, knowledge gaps, and context-specific contributions of urban gardening initiatives to sustainable development, particularly in the Global South.

The following five core analytical themes guided the data synthesis:

1. General Study Characteristics
  - Geographical scope: Country, region, and urban setting (e.g., city or town context).
  - Urban typology: Type of urban garden studied (e.g., home gardens, rooftop gardens, community gardens, school gardens).
  - Study design: Qualitative, quantitative, or mixed-methods approaches.
  - Temporal scope: Duration of study, seasonality considerations, and longitudinal relevance.
  - Data collection methods: Interviews, field surveys, remote sensing, participatory methods, policy analysis, or experimental plots.
2. Ecological and Biodiversity Contributions
  - Plant species composition and diversity: Indigenous vs. exotic species, functional traits, and ecosystem services.
  - Urban ecology outcomes: Pollinator support, urban biodiversity enrichment, soil health, and microclimate regulation.
  - Integration with green infrastructure: Linkages to parks, street trees, green corridors, and land use planning.
3. Technological and Management Aspects
  - Use of modern technologies: Smart irrigation, vertical farming, hydroponics, GIS-based planning.
  - Innovation in design and implementation: Mobile garden units, recycled materials, adaptive reuse of urban spaces.
  - Maintenance practices and community participation models.
4. Socio-Economic and Community Impacts
  - Food security outcomes: Crop diversity, nutritional benefits, household food provisioning.
  - Livelihoods and economic opportunities: Income generation, local enterprise development, job creation.
  - Health and well-being: Mental health, physical activity, and social cohesion indicators.
  - Equity and inclusiveness: Gender participation, access by marginalised groups, and affordability.
5. Urban Planning and Governance Interface
  - Policy integration: How urban gardens are incorporated into local planning frameworks, municipal strategies, and land-use policies.
  - Planning instruments: Zoning laws, incentives for green space development, and spatial inclusion of food production zones.
  - Barriers and enablers: Institutional, infrastructural, or socio-political constraints and drivers of urban gardening adoption.

## 5. Conclusions

Urban gardening plays a pivotal role in promoting ecological sustainability, food security, and social resilience across African cities, yet its strategic integration into urban development frameworks remains limited. This review highlights that while ornamental and indigenous plant species are present in urban gardens, their selection is often guided more by socio-economic factors than by clear ecological or planning criteria. Analysis of the reviewed studies further reveals that certain crop families dominate urban gardening systems, for example, Fabaceae (legumes), Solanaceae (tomatoes, peppers, eggplants), Poaceae (*Zea mays*), and Asteraceae (leafy vegetables and culturally important species). These groups are favoured for their combined nutritional, cultural, and ecological benefits, including dietary protein provision, pollinator support, and soil fertility enhancement. Recognising the multifunctionality of these dominant crops alongside ornamental species offers a strong entry point for integrated planning.

The underreporting of species selection rationales, particularly in relation to ecological functionality, cultural significance, and microclimate regulation, reflects a critical gap in both research and policy. To strengthen the role of ornamental plants in urban gardening systems, city planners and policymakers must recognise these spaces as multifunctional green infrastructure rather than informal or transitional land use.

From a policy perspective, our synthesis of crop family diversity offers a reference point for developing urban gardening plant selection guidelines that balance nutritional value, ecological resilience, and cultural relevance. Municipal and national policies could integrate these insights into urban gardening initiatives, ensuring that promoted plant lists reflect both community needs and biodiversity conservation goals. Zoning regulations should formally allocate land for urban gardening, particularly in underserved communities, and policy frameworks should incentivise plant choices that balance ornamental value with ecological and socio-economic functions.

Practically, these findings suggest that urban gardening can be scaled as a low-cost, multifunctional intervention to address pressing urban challenges such as food insecurity, unemployment, and climate vulnerability. Guidelines for species diversification, pollinator-friendly plantings, and environmentally resilient ornamentals that are developed in collaboration with local communities, horticulturists, and ecologists could ensure that planting schemes are both aesthetically enriching and climate-adaptive. Public investment in community nurseries, mobile advisory services, and participatory greening initiatives can further strengthen urban gardening as a tool for social inclusion and ecological resilience. As African cities confront climate change, urban sprawl, and food insecurity, aligning species selection with nutrition-sensitive and biodiversity-enhancing policies offers an untapped opportunity to transform urban gardens into resilient, culturally meaningful, and socially inclusive spaces.

## Figures and Tables

**Table 1:** Boolean search terms and operators used in the literature review.

Concept	Search Terms	Notes
Urban gardening and agriculture	(“urban garden” OR “urban agriculture” OR “community garden” OR “home garden’’ OR “food garden” OR “rooftop garden” OR “vertical garden” OR “domestic garden” OR “private green space”)	Core topic—ensures inclusion of all types of urban gardening activities
Food security, biodiversity and climate change	(“food security” OR “urban food system” OR “climate change” OR “climate resilience” OR “food sovereignty” OR “plant selection” OR “urban biodiversity”)	Targets thematic alignment with SDG goals and environmental drivers
Socio-economic resilience	(“livelihoods” OR “economic resilience” OR “social capital” OR “community empowerment”)	Focuses on the societal dimensions and community benefits of urban gardens
Sustainable urban development	(“sustainable city” OR “urban development” OR “resilient city” OR “urban sustainability”)	Captures planning and sustainability literature
Urban planning and policy	(“city planning” OR “urban planning” OR “land use policy” OR “zoning” OR “municipal development plan” OR “green space”)	Introduces city-scale governance and integration of urban gardens into urban design and planning
Technological interventions	(“smart technology” OR “modern technology” OR “smart agriculture” OR “smart agricultural technology” OR “climate-smart technology” OR “hydroponics” OR “aquaponics” OR “smart sensors”)	Introduces the use and implementation of modern technologies in urban gardening
Regional focus	(“Sub-Saharan Africa” OR “developing countries” OR “global south” OR “Africa”)	Ensures geographical relevance to urban areas in need of development focus

## Data Availability

The data supporting the findings of this study are derived from previously published sources, which are cited throughout the article. The systematic review protocol is publicly available on Protocols.io. The data extraction sheets are available from the authors upon request.

## Abbreviations

The following abbreviations are used in this manuscript:

CSATs	Climate-smart agricultural technologies
EBHAF	Enset-based home garden agroforestry
GIS	Geographic Information Systems
OECD	Organization for Economic Co-operation and Development
PAF	Parkland agroforestry
